# Phenotypic and Functional Signatures of Peripheral Blood and Spleen Compartments of Cynomolgus Macaques Infected With *T. cruzi*: Associations With Cardiac Histopathological Characteristics

**DOI:** 10.3389/fcimb.2021.701930

**Published:** 2021-07-14

**Authors:** Renato Sathler-Avelar, Danielle Marquete Vitelli-Avelar, Armanda Moreira Mattoso-Barbosa, Marcelo Antônio Pascoal-Xavier, Silvana Maria Elói-Santos, Ismael Artur da Costa-Rocha, Andréa Teixeira-Carvalho, Edward J. Dick, Jane F. VandeBerg, John L. VandeBerg, Olindo Assis Martins-Filho

**Affiliations:** ^1^ Instituto René Rachou, FIOCRUZ-Minas, Belo Horizonte, Brazil; ^2^ Southwest National Primate Research Center, Texas Biomedical Research Institute, San Antonio, TX, United States; ^3^ Faculdade de Minas, FAMINAS-BH, Belo Horizonte, Brazil; ^4^ Faculdade de Ciências Médicas de Minas Gerais, FCMMG, Belo Horizonte, Brazil; ^5^ Departamento de Propedêutica Complementar, Faculdade de Medicina, Universidade Federal de Minas Gerais, Belo Horizonte, Brazil; ^6^ South Texas Diabetes and Obesity Institute and Department of Human Genetics, School of Medicine, The University of Texas Rio Grande Valley, Brownsville/Harlingen/Edinburg, TX, United States; ^7^ Center for Vector-Borne Diseases, The University of Texas Rio Grande Valley, Brownsville/Harlingen/Edinburg, TX, United States

**Keywords:** non-human primates, cynomolgus macaques, *Trypanosoma cruzi*, cardiac Chagas disease, immune response, cytokines

## Abstract

We performed a detailed analysis of immunophenotypic features of circulating leukocytes and spleen cells from cynomolgus macaques that had been naturally infected with *Trypanosoma cruzi*, identifying their unique and shared characteristics in relation to cardiac histopathological lesion status. *T. cruzi-*infected macaques were categorized into three groups: asymptomatic [CCC(-)], with mild chronic chagasic cardiopathy [CCC(+)], or with moderate chronic chagasic cardiopathy [CCC(++)]. Our findings demonstrated significant differences in innate and adaptive immunity cells of the peripheral blood and spleen compartments, by comparison with non-infected controls. CCC(+) and CCC(++) hosts exhibited decreased frequencies of monocytes, NK and NKT-cell subsets in both compartments, and increased frequencies of activated CD8^+^ T-cells and GranA^+^/GranB^+^ cells. While a balanced cytokine profile (TNF/IL-10) was observed in peripheral blood of CCC(-) macaques, a predominant pro-inflammatory profile (increased levels of TNF and IFN/IL-10) was observed in both CCC(+) and CCC(++) subgroups. Our data demonstrated that cardiac histopathological features of *T. cruzi*-infected cynomolgus macaques are associated with perturbations of the immune system similarly to those observed in chagasic humans. These results provide further support for the validity of the cynomolgus macaque model for pre-clinical research on Chagas disease, and provide insights pertaining to the underlying immunological mechanisms involved in the progression of cardiac Chagas disease.

## Introduction

Chagas disease is caused by the flagellate protozoan *T. cruzi*, and affects approximately 6-7 million people worldwide (World Health Organization, 2020). The disease typically has a short acute phase, which may or may not proceed to a long-lasting chronic disease. Most individuals with chronic Chagas disease remain asymptomatic, but 20 to 40% of patients develop clinical illness with digestive or cardiac pathologies (Prata, 1990; Dias, 1995; Rassi et al., 2010). The prognosis of patients who have cardiac disease caused by *T. cruzi* infection is worse than that of patients whose cardiac disease has other etiologies, and frequently leads to death from sudden cardiac arrest (Bocchi et al., 2017; Bonney et al., 2019).

Several ethiopathogenic bases have been proposed for the cardiac damage caused by Chagas disease, including direct parasite-induced damage, neurogenic events, microvascular circulation disorders and inflammatory/autoimmune tissue injury (Higuchi et al., 2003; Bocchi et al., 2017). Some studies have shown that parasite genotype and host genetic background may be associated with distinct clinical manifestations of Chagas disease (Macedo and Pena, 1998; Macedo et al., 2004).

Chronic Chagas cardiomyopathy (CCC) may involve a complex variety of immunological events leading to distinct histopathological features (Reis et al., 1993; Higuchi et al., 2003; Fonseca et al., 2007). Many specific cell populations and cytokines involved in the immunopathological mechanisms underlying cardiac Chagas disease have been identified (Reis et al., 1993; Higuchi et al., 2003; Fonseca et al., 2007; Costa et al., 2009; Cunha-Neto and Chevillard, 2014; Ferreira et al., 2014; Roffe et al., 2019). Despite the many well-described immunological factors involved in CCC, their hypothetical interactions that might lead to different cardiac pathologies are still not well understood. Additional characterization of the immunological events that take place in distinct compartments where immunity cells congregate may contribute to a better understanding of the interactions involved in the multifactorial nature of myocarditis caused by Chagas disease. In this regard, analysis of distinct immunological compartments in experimental models that exhibit histopathological and immunological characteristics likewise those observed in human Chagas disease may provide novel insights.

Here, we present a comprehensive assessment of several phenotypic and functional immune characteristics in peripheral blood leukocytes and spleen cells from cynomolgus macaques that had become naturally infected with *T. cruzi*, aiming to determine their unique and shared characteristics in relation to cardiac histopathological disease status. Our findings demonstrated significant alterations in innate and adaptive immunity cells of peripheral blood and spleen compartments of *T. cruzi*-infected macaques by comparison with non-infected animals. The results provide further confirmation of the similarities between cynomolgus macaques naturally infected with *T. cruzi* and humans with Chagas disease. Our detailed assessment of immunological events associated with distinct patterns of chagasic cardiac disease provides a basis for further pre-clinical research with non-human primate models, as well as for clinical research with human subjects.

## Material and Methods

### Study Population and Ethics Statement

Twenty-six cynomolgus macaques were enrolled in this cross-sectional investigation. The diagnosis of natural *T. cruzi* infection was performed by serological tests, including anti-*T. cruzi* antibody detection by enzyme-linked immunoassay (ELISA) and immuno-chromatographic assay. All animals were infected with TcI genotype, identified by molecular characterization of *T. cruzi* isolated from peripheral blood samples as previously described (Vitelli-Avelar et al., 2017). The group of naturally infected macaques (CH for “Chagas”, n=15) comprised 3 males and 12 females with median body weight of 3.5kg and median age of 12 years. All CH macaques were in the chronic phase of disease determined by the absence of patent parasitemia and further supported by histopathological analysis performed during necropsies. Based on cardiac histopathologic features, the CH macaques were divided into three subgroups: asymptomatic [CCC(-), n=5], mild chronic chagasic cardiopathy [CCC(+), n=4] and moderate chronic chagasic cardiopathy [CCC(++), n=6]. Although the duration of time that each animal was infected with the parasite is not known, most of these animals were young to middle-aged adults. A 12-year-old macaque is approximately equivalent in age to a 36-year-old human. Therefore, the absence of severe chagasic cardiopathy among the 15 infected macaques may be a consequence of insufficient duration of infection to have resulted in severe cardiac pathology. These macaques were group-housed and highly active in their social environment, it is likely that animals with severe cardiac pathologies died spontaneously from acute cardiac episodes or were culled as a consequence of poor health status. The non-infected controls (NI, n=11), which included 2 males and 9 females with a median body weight of 4.9kg and a median age of 13 years, displayed negative results in both serological tests.

This study was approved by the Texas Biomedical Research Institute Animal Care and Use Committee (#1050MF), and was conducted in accordance with the Public Health Service Policy on Humane Care and Use of Laboratory Animals, and the U.S. Animal Welfare Act. Animal care was provided according to the Guide for the Care and Use of Laboratory Animals. The animals received commercial chow and water *ad libitum*, supplemented with fruits and vegetables.

### Biological Samples

Ten mL of heparinized blood was drawn from the femoral vein after general anesthesia by intra-muscular injection of ketamine hydrochloride (10mg/kg) and valium (5mg/kg), and inhalation of isofluorane (1.5%). The blood samples were used for *ex vivo* immunophenotypic and functional analysis by flow cytometry.

Spleen specimens were collected from each animal during necropsy, and splenocytes were isolated for *ex vivo* immunophenotypic analysis by flow cytometry. The specimens were immersed in cold RPMI‐1640 in a Petri dish placed on ice. The spleen fragments were processed according to Teixeira-Carvalho et al. (2002). Heart specimens also were collected during necropsy for histopathologic analysis.

### *Ex Vivo* Immunophenotypic Analysis of Peripheral Blood and Spleen Cells by Flow Cytometry

Immunophenotypic analysis of leukocytes and spleen cells was carried out as follows: aliquots of heparinized whole peripheral blood (100μL) or splenocyte suspensions (5x10^5^ cells in 50μL) were incubated with mixtures of undiluted fluorescent labeled monoclonal antibodies (5μL) for 30 min at room temperature, in the dark. Mouse monoclonal antibodies, specific for human cell surface markers and with cross-reactivity to the same non-human primate markers, were used in this study: FITC α-CD4, α-CD14, α-CD16, α-CD32, α-CD64, α-GranA, α-GranB and α-Perforin; PE α-CD4, α -CD14, α-CD54, α-CD56, α-CCR5, α-CD25 and α-CD69; PerCP-Cy5.5 α-CD4, α-CD8 and α-HLA-DR; APC α-CD8, α-CD16 and α-CD20; Alexa fluor 700 α-CD3. After incubation, erythrocytes were lysed by adding 2mL of Lysing Solution, followed by incubation for 10 min at room temperature, in the dark. The stained leukocyte and splenocyte suspensions were washed twice with phosphate-buffered saline (PBS) supplemented with 0.01% sodium azide. Cells were then fixed with 200μL of Fixing Solution (10g/L of paraformaldehyde, 10.2g/L of sodium cacodylate, 6.65 g/L of sodium-chloride, pH 7.2) and stored at 4°C.

Intracellular staining was performed to quantify GranA^+^, GranB^+^ and Perf^+^ within CD16^+^ and CD8^+^ cells. Aliquots of blood (100μL) or splenocyte suspensions (5x10^5^ cells in 50μL) were first incubated with 5μL of anti-CD16 or anti-CD8, for 30 min at room temperature, in the dark. Following erythrocyte lysis and pre-fixation with Lysing Solution, the remaining cells were permeabilized with 2mL of Perm-Buffer (PBS supplemented with 0.5% bovine serum albumin, 0.5% saponin, 0.01% sodium azide), for 10 min at room temperature, in the dark. Fixed/permeabilized cells were then incubated with 5μL of anti-GranA, anti-GranB or anti-Perf for 30 min at room temperature, in the dark. Stained cells were washed once with Perm-Buffer and then with Buffer (PBS supplemented with 0.5% bovine serum albumin, 0.01% sodium azide) and fixed in 200μL of Fixing Solution and stored at 4°C.

A total of 30,000 events were acquired per sample using a CyAn-ADP Flow Cytometer. Summit software 4.3.01 was used for data acquisition and analysis. The FlowJo software 9.4.1 was used for data analysis. Distinct gating strategies were employed to select specific cell populations. Monocytes were first gated as CD14^High+^ cells with subsequent analysis of CD32 and CD64 expression. After lymphocyte gating, combined gating strategies were used to analyze NK (CD3^-^CD16^+^), NKT (CD3^+^CD16^+^), T (CD3^+^, CD4^+^, CD8^+^) and B-cells (CD20^+^) for subsequent analysis of complementary immunophenotypic features. The results were expressed as percentage (%) or mean fluorescence intensity (MFI) within specific whole blood or spleen cell subsets. Representative flow cytometric gating strategies employed for phenotypic analysis are provided in the **Supplementary Figure 1**.

### *Ex Vivo* Functional Analysis of Peripheral Blood Cells by Flow Cytometry

The *ex vivo* functional analysis of peripheral blood leukocytes was performed as described previously by Vitelli-Avelar et al. (2008). Briefly, 500μL aliquots of heparinized whole blood samples were diluted 1:1 with 500μL of RPMI-1640 plus Brefeldin-A at a final concentration of 10μg/mL and incubated in triplicate for 4 hours at 37°C, in a 5% CO2 humidified atmosphere. Following incubation, cells were treated with 100μL of 200mM EDTA, and then incubated at room temperature for 10 min. The triplicates were pooled prior to immunostaining for intracytoplasmic cytokine analysis by flow cytometry. The immunostaining procedure was carried out as described previously by Vitelli-Avelar et al. (2008). Briefly, cells were washed once and re-suspended in Buffer, and 100μL aliquots were transferred to 5mL polystyrene tubes containing 5µL of FITC or TC monoclonal antibodies (α-CD14, α-HLA-DR, α-CD16, α-CD3, α-CD4, α-CD8 or α-CD20). Following incubation in the dark for 30 min at room temperature, erythrocytes were lysed with 2mL of Lysing Solution, and the remaining cells were fixed in Fixing Solution for 10 min at room temperature, in the dark. The membrane-stained fixed cells were permeabilized with Perm-Buffer for 30 min at room temperature, in the dark. Fixed/Permeabilized cells were then incubated with 5μL of PE-labeled monoclonal antibodies (α-TNF, α-IFN-γ and α-IL-10) for 30 min at room temperature, in the dark. Stained cells were washed once with Perm-Buffer and then with Buffer prior to fixation with 200μL of Fixing Solution. Cells were stored at 4°C.

A total of 30,000 events were acquired per sample using a CyAn-ADP Flow Cytometer. Summit software 4.3.01 was used for data acquisition and analysis. Monocytes were gated as CD14^High+^ cells followed by CD16^+^/HLADR^++^ events, with subsequent analysis of cytokine^+^ cells (TNF^+^ and IL-10^+^). Combined gating strategies were used to analyze NK, T and B-cells. After gating, CD16^+^, CD3^+^, CD4^+^, CD8^+^ or CD20^+^ were gated for subsequent analysis of cytokine^+^ cells. The results were expressed as per-mille (‰) of cytokine^+^ cells within specific cell subsets. Representative flow cytometric gating strategies employed for functional analysis are provided in the **Supplementary Figure 1**.

### Heart Histopathological Analysis

The heart specimens used for histopathological analysis were fixed in 10% neutral buffered formalin, dehydrated in alcohol, cleared in xylene and embedded in paraffin blocks. Sections of 5µm thickness were cut and stained with hematoxylin and eosin.

Histopathological analysis was carried out by (MAXP), using conventional optical microscopy. The histopathological findings were categorized in relation to the distribution and intensity of inflammatory infiltrates. The Distribution of Inflammatory Infiltrate (DII) was classified as absent/focal (0) or multifocal/diffuse (1), and the Intensity of Inflammatory Infiltrate (III) was classified as basal (0) or elevated (1). Based on the histopathological results, the *T. cruzi*-infected macaques were categorized into three subgroups, referred as: asymptomatic [CCC(-), n=5; DII(0) and III(0)], mild chronic chagasic cardiopathy [CCC(+), n=4; DII(1) and III(0)] and moderate chronic chagasic cardiopathy [CCC(++), n=6; DII(1) and III(1)].

### Data Analysis

Descriptive statistical analyses were performed for multiple comparisons among groups using the Kruskal-Wallis test followed by Dunn’s post-test for sequential pairwise comparisons. GraphPad Prism Software 6.0 was used for descriptive statistical analysis. In all cases, significant differences were considered at p<0.05.

**Figure 1:**
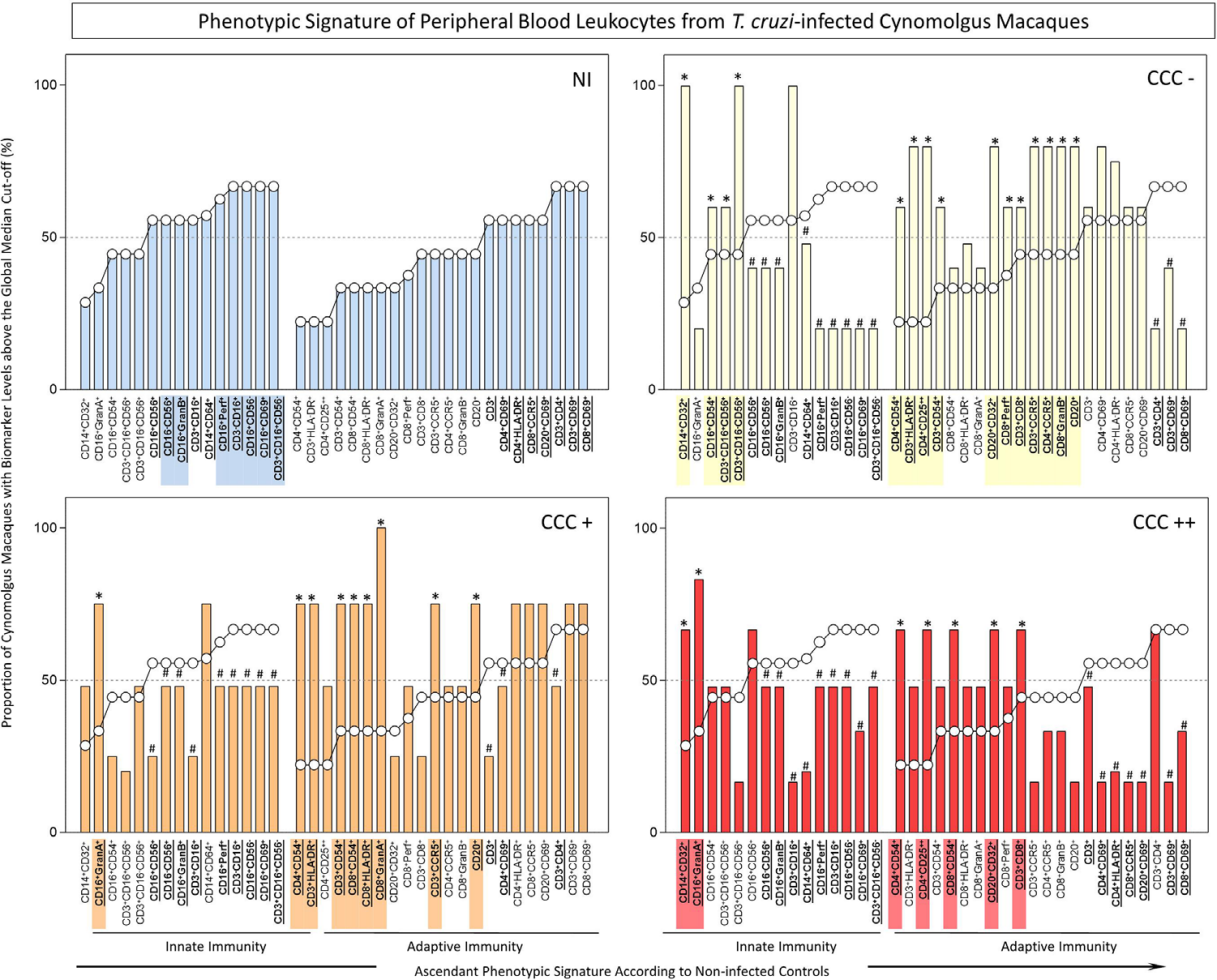
*Ex-vivo* phenotypic signatures of peripheral blood leukocytes from *T. cruzi*-infected cynomolgus macaques classified according to histopathological features of chronic chagasic cardiopathy. The phenotypic signatures were constructed based on the proportion of subjects in each cell subpopulation with biomarker levels above the global median cut-off, calculated from data from the entire study population. Ascendant curves were assembled from non-infected controls (NI) to draw the reference curves for innate and adaptive immunity cells, used for comparative analysis of results from *T. cruzi*-infected cynomolgus macaques. Data are displayed by bar charts (NI = 
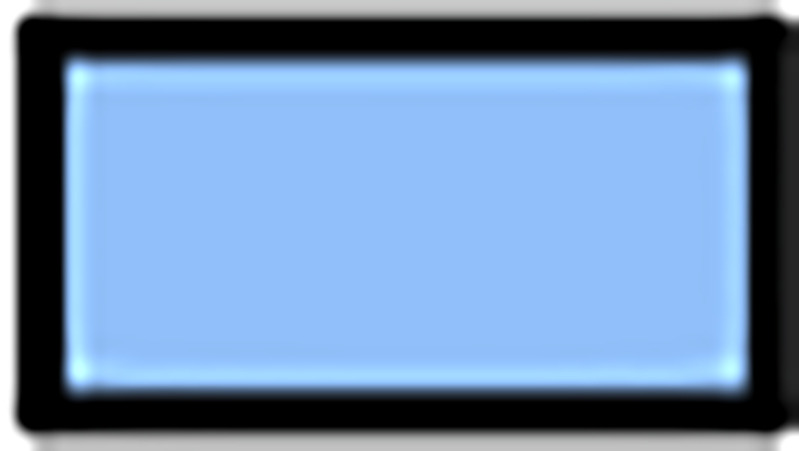
 ) and continuous ascendant lines (NI = ○). The *T. cruzi*-infected macaques were classified according to histopathological features of cardiac biopsies and referred to as CCC(–) for absence of chronic chagasic cardiopathy (
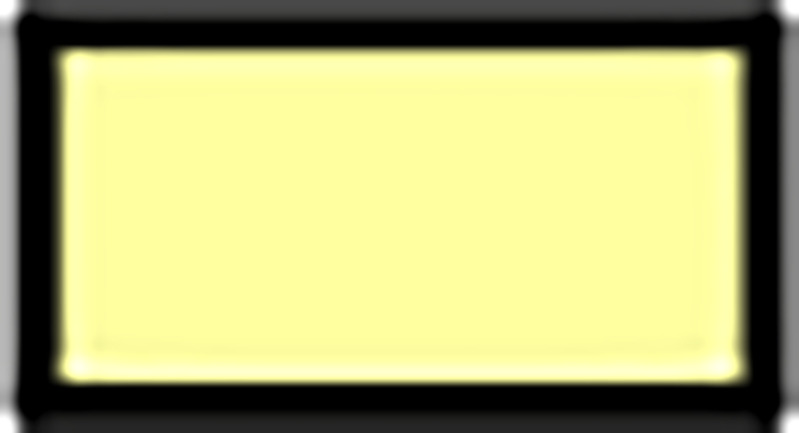
); CCC(+) for mild chronic chagasic cardiopathy (
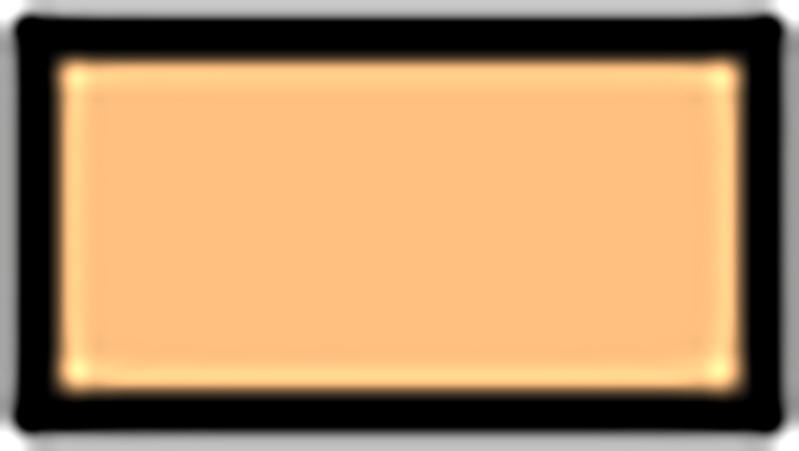
) and CCC(++) for moderate chronic chagasic cardiopathy (
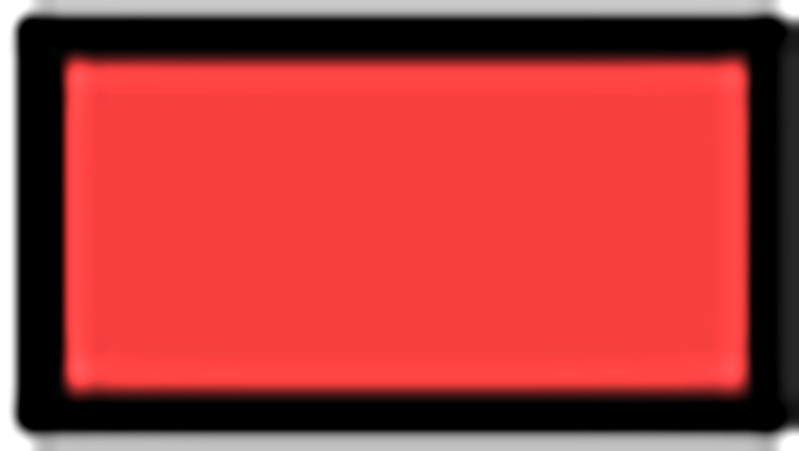
). Comparative analyses between *T. cruzi*-infected subgroups and non-infected controls were carried out considering the ascendant biomarker signature of non-infected controls as the reference curve. The differences between groups were considered for biomarkers with proportions confined to distinct 50^th^ percentiles as compared to the reference curve. The biomarkers with lower frequencies in *T. cruzi*-infected macaques were underscored by # and bold underline format. Those biomarkers with higher frequencies in *T. cruzi*-infected macaques were highlighted by *, bold underline format and color background.

**Figure 2:**
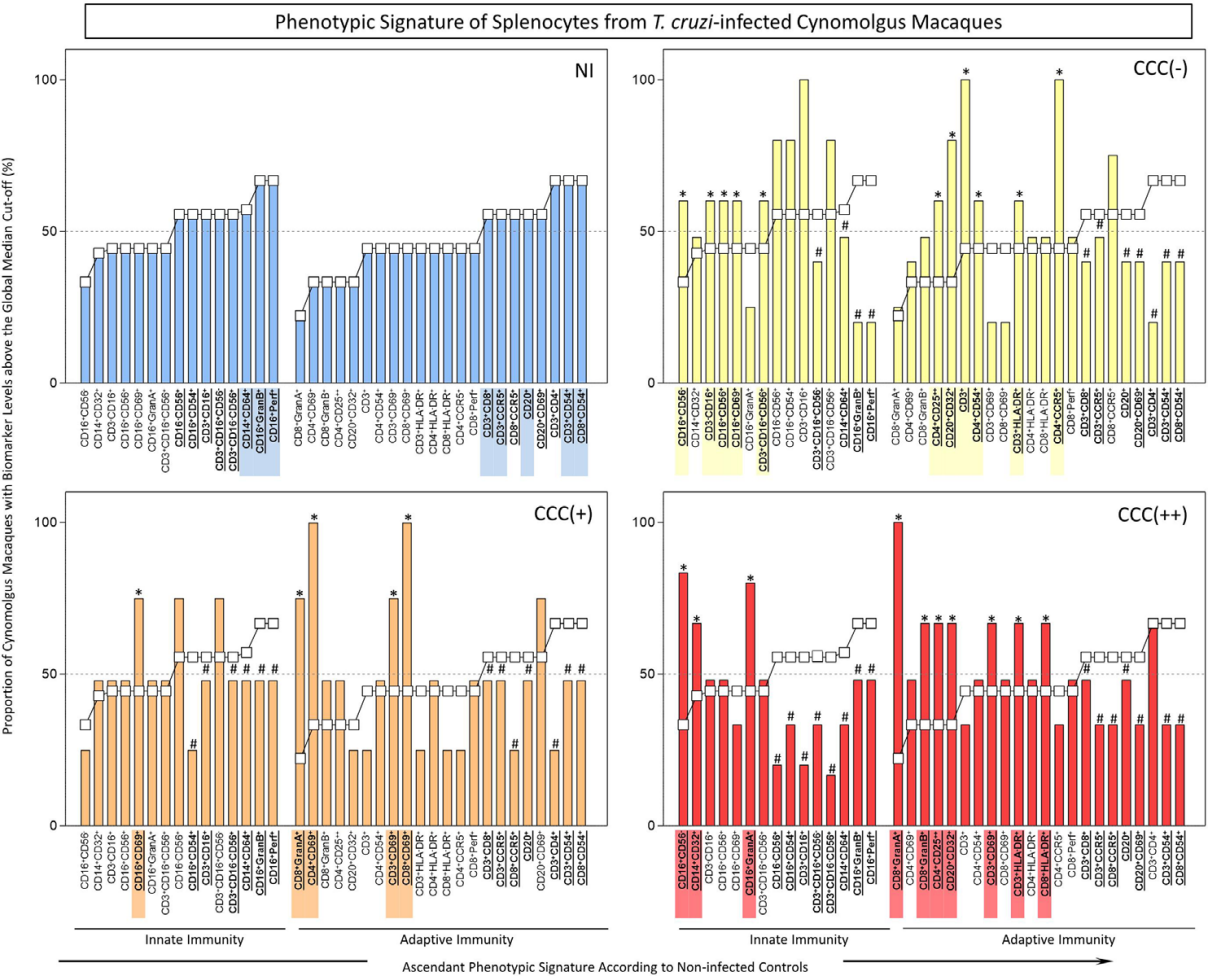
*Ex-vivo* phenotypic signatures of splenocytes from *T. cruzi*-infected cynomolgus macaques classified according to histopathological features of chronic chagasic cardiopathy. See legend to **Figure 1** for details.

Additionally, analysis of phenotypic signatures of peripheral blood leukocytes and splenocytes, as well as the cytokine signatures of peripheral blood leukocytes, were carried out by first converting the *ex vivo* immunophenotypic results from continuous variables into categorical data. The global median values of each cell phenotype were calculated from the values of all 26 macaques. Phenotypic signatures of the innate immunity biomarkers and the adaptive immunity biomarkers in the NI group were developed by creating bar graphs showing, for each biomarker, the proportion of animals with values higher than the global median, arranged with the biomarkers in ascending order of those values (see **Figures 1** and **2**, graphs labeled NI). Bar graphs for the biomarkers of the three infected groups were developed with the biomarkers arranged in the same orientation as for the NI group, and statistical analyses were conducted to identify significant differences between the NI group and each of the other groups. Radar charts were used to compile the cytokine signatures of peripheral blood leukocytes of *T. cruzi*-infected cynomolgus macaques and NI controls. The biomarkers with frequencies significantly above the global 50^th^ percentile were highlighted for each group for comparative analysis. Venn diagram analysis were performed to identify sets of common and unique biomarkers for comparisons of CCC(–) *vs* CCC(+) *vs* CCC(++) *vs* NI, using online software, available at http://bioinformatics.psb.ugent.be/webtools/Venn/.

Graphical arts were performed using Microsoft Excel and GraphPad Prism Softwares.

## Results

### Analyses of Peripheral Blood Leukocytes From *T. cruzi*-Infected Cynomolgus Macaques Classified According to Histopathological Features of Chronic Chagasic Cardiopathy

Detailed flow cytometric results expressed as percentage or mean fluorescence intensity (MFI), along with descriptive statistical analysis for the phenotypic features of peripheral blood leukocytes from *T. cruzi*-infected macaques, by comparison with non-infected controls, are presented in **Table 1**. Data analysis demonstrated significant differences in phenotypic features of innate (NK and NKT-cell subsets) and adaptive immunity (CD4^+^ and CD8^+^ T-cells) of *T. cruzi*-infected cynomolgus macaques as compared to non-infected controls. Of note, higher levels of GranA^+^ NK-cells and CD8^+^ T-cells were observed in hosts with mild/CCC(+) or moderate/CCC(++) chronic chagasic cardiopathy ad compared to asymptomatic/CCC(-) hosts (**Table 1**).

**Table 1:** Phenotypic features of peripheral blood and spleen leukocytes from *T.* cruzi-infected cynomolgus macaques.

Cell Phenotypes	Peripheral Blood				Spleen			
	NI	CCC(-)	CCC(+)	CCC(++)	NI	CCC(-)	CCC(+)	CCC(++)
Innate Immunity								
CD14^+^CD32^+^ (MFI)	304.8 ± 41.8	411.3 ± 59.7	445.8 ± 99.8	320.2 ± 72.7	459.4 ± 55.2	559.5 ± 67.8	475.3 ± 73.7	490.8 ± 19.2
CD14^+^CD64^+^ (MFI)	65.6 ± 9.0	92.1 ± 39.9	78.0 ± 21.0	41.8 ± 5.9	89.8 ± 11.2	102.6 ± 22.6	95.7 ± 26.1	91.8 ± 26.1
CD3^-^CD16^+^	9.0 ± 2.0	4.9 ± 1.5	11.1 ± 3.2	12.4 ± 4.5	16.6 ± 4.6	12.3 ± 2.7	16.3 ± 5.9	15.8 ± 4.8
%CD16^+^CD56^-^	14.1 ± 3.4	**5.3 ± 1.4^a,c,d^**	10.8 ± 2.6	16.3 ± 6.0	27.7 ± 7.7	21.8 ± 5.0	28.9 ± 11.7	34.5 ± 6.8
%CD16^+^CD56^+^	5.9 ± 1.4	6.1 ± 3.8	7.6 ± 2.9	10.8 ± 4.4	3.4 ± 0.4	5.9 ± 1.3	4.4 ± 0.4	4.3 ± 0.4
%CD16^-^CD56^+^	6.2 ± 0.6	6.4 ± 1.2	6.9 ± 2.4	5.2 ± 1.1	5.5 ± 0.3	7.7 ± 0.9	7.2 ± 1.2	4.1 ± 0.5
%CD16^+^CD54^+^	8.8 ± 1.7	15.6 ± 4.0	7.6 ± 3.8	8.3 ± 2.7	16.1 ± 2.4	26.5 ± 4.5	12.9 ± 5.7	13.5 ± 1.8
%CD16^+^CD69^+^	35.5 ± 5.9	**22.2 ± 3.1^a,c^**	34.8 ± 6.0	26.5 ± 7.5	32.3 ± 2.8	32.3 ± 4.1	43.2 ± 8.7	29.6 ± 9.3
%CD16^+^GranA^+^	16.6 ± 3.6	**14.3 ± 2.8^a^**	**38.1 ± 11.2^a,b^**	**40.3 ± 9.6^a,b^**	22.0 ± 2.5	21.1 ± 3.1	33.0 ± 7.8	37.5 ± 5.3
%CD16^+^GranB^+^	45.7 ± 3.9	36.1 ± 5.8	45.4 ± 12.4	47.0 ± 9.2	59.1 ± 3.6	34.5 ± 3.0	52.6 ± 6.1	53.1 ± 6.6
%CD16^+^Perf^+^	46.1 ± 6.6	37.8 ± 4.0	52.0 ± 9.1	46.9 ± 6.5	60.9 ± 3.7	31.9 ± 2.4	54.9 ± 5.4	52.8 ± 6.2
CD3^+^CD16^+^	3.5 ± 0.4	4.2 ± 0.1	**2.6 ± 0.8^b^**	**3.2 ± 0.7^b^**	7.3 ± 0.9	8.2 ± 0.6	7.1 ± 1.2	4.7 ± 0.4
% CD3^+^CD16^+^CD56^-^	2.6 ± 0.7	**0.9 ± 0.2^a^**	0.9 ± 0.1	1.7 ± 0.6	5.0 ± 0.6	4.6 ± 1.2	5.2 ± 0.6	5.5 ± 1.7
% CD3^+^CD16^+^CD56^+^	0.1 ± 0.0	0.1 ± 0.0	0.1 ± 0.0	0.4 ± 0.2	1.3 ± 0.2	1.2 ± 0.2	1.5 ± 0.6	1.2 ± 0.3
% CD3^+^CD16^-^CD56^+^	3.6 ± 0.3	**5.8 ± 0.3^a,c,d^**	3.6 ± 0.7	2.7 ± 0.4	6.3 ± 0.7	6.9 ± 0.9	4.9 ± 0.4	3.8 ± 1.1
Adaptive Immunity								
CD3^+^	62.0 ± 3.4	60.5 ± 3.1	58.1 ± 7.0	64.0 ± 5.2	54.6 ± 4.6	64.1 ± 2.6	51.6 ± 6.7	51.1 ± 6.0
CD3^+^CD4^+^	32.7 ± 2.5	**23.0 ± 2.8^a^**	**28.7 ± 1.3^d^**	**32.7 ± 1.2^b^**	16.2 ± 1.8	13.7 ± 2.5	12.9 ± 1.6	16.7 ± 3.3
CD3^+^CD8^+^	31.1 ± 3.3	37.4 ± 5.7	28.7 ± 8.7	35.9 ± 5.0	40.2 ± 4.3	38.1 ± 7.7	36.0 ± 5.7	33.6 ± 6.1
%CD3^+^CD54^+^	1.0 ± 0.1	**2.7 ± 0.9^a^**	2.3 ± 1.0	1.4 ± 0.3	5.3 ± 0.8	4.6 ± 0.5	6.2 ± 1.6	3.3 ± 0.7
%CD4^+^CD54^+^	1.4 ± 0.2	**3.7 ± 1.2^a^**	17.8 ± 15.7	6.9 ± 1.4	7.5 ± 1.0	9.5 ± 1.9	11.9 ± 4.4	6.4 ± 1.4
%CD8^+^CD54^+^	1.2 ± 0.2	**2.9 ± 1.5^a^**	**8.5 ± 4.2^a^**	**2.9 ± 1.0^a^**	3.9 ± 0.5	4.1 ± 0.9	4.2 ± 0.9	3.2 ± 0.7
%CD3^+^CD69^+^	9.6 ± 1.9	8.5 ± 2.8	9.7 ± 1.9	5.7 ± 1.6	26.1 ± 2.8	26.5 ± 3.2	36.0 ± 3.0	31.7 ± 4.2
%CD4^+^CD69^+^	4.2 ± 1.0	4.5 ± 1.5	4.3 ± 1.5	3.2 ± 0.7	25.5 ± 2.1	30.0 ± 3.6	34.6 ± 1.6	28.7 ± 2.7
%CD8^+^CD69^+^	23.1 ± 4.0	16.1 ± 2.5	**26.3 ± 4.7^b,d^**	16.1 ± 2.5	24.3 ± 2.5	27.0 ± 5.2	36.3 ± 2.1	30.2 ± 4.9
%CD3^+^HLA-DR^+^	3.1 ± 0.7	**4.2 ± 0.7^a^**	**5.6 ± 1.3^a,d^**	3.0 ± 0.3	6.6 ± 0.8	9.0 ± 0.6	8.3 ± 1.1	11.0 ± 1.4
%CD4^+^HLA-DR^+^	3.1 ± 0.4	4.6 ± 1.3	3.9 ± 0.6	**2.2 ± 0.2^c,b^**	18.1 ± 1.7	20.6 ± 5.3	19.5 ± 4.4	17.4 ± 3.0
%CD8^+^HLA-DR^+^	5.6 ± 1.8	7.7 ± 1.8	7.8 ± 1.4	6.5 ± 1.6	6.8 ± 0.9	7.3 ± 1.8	6.3 ± 1.9	10.4 ± 1.6
%CD3^+^CCR5^+^	26.4 ± 4.6	29.2 ± 7.5	28.7 ± 2.7	20.6 ± 5.9	33.0 ± 4.0	39.6 ± 9.1	29.1 ± 6.0	30.0 ± 5.9
%CD4^+^CCR5^+^	19.7 ± 2.9	24.8 ± 4.6	16.3 ± 1.2	17.5 ± 3.8	34.6 ± 2.7	48.7 ± 6.8	30.2 ± 2.8	29.7 ± 4.9
%CD8^+^CCR5^+^	31.6 ± 5.5	34.5 ± 7.3	38.0 ± 4.5	25.3 ± 7.5	30.6 ± 3.7	41.8 ± 8.0	25.8 ± 5.5	23.2 ± 4.8
%CD8^+^GranA^+^	6.9 ± 4,5	**9.1 ± 1.2^a^**	**15.8 ± 2.8^a,b^**	**15.9 ± 3.5^a,b^**	11.0 ± 1.9	11.2 ± 2.6	14.9 ± 1.7	17.5 ± 1.9
%CD8^+^GranB^+^	52.6 ± 7.2	58.2 ± 11.9	49.0 ± 9.1	55.9 ± 7.4	43.6 ± 5.5	43.6 ± 8.5	38.8 ± 8.7	46.8 ± 3.8
%CD8^+^Perf^+^	56.2 ± 7.9	69.1 ± 11.0	62.7 ± 8.1	63.3 ± 8.0	58.4 ± 6.6	61.3 ± 9.6	58.1 ± 9.1	57.6 ± 6.1
%CD4^+^CD25^++^	3.5 ± 0.3	4.1 ± 0.5	4.1 ± 0.5	4.3 ± 0.3	2.4 ± 0.4	3.0 ± 0.6	2.7 ± 0.7	3.8 ± 0.7
CD20^+^	22.4 ± 4.7	27.1 ± 3.5	28.7 ± 6.3	19.9 ± 4.5	22.4 ± 4.5	19.6 ± 2.7	20.0 ± 6.7	26.8 ± 6.4
%CD20^+^CD69^+^	2.6 ± 0.4	3.5 ± 1.1	3.3 ± 0.5	2.6 ± 1.2	13.9 ± 1.8	13.2 ± 2.2	19.5 ± 2.9	13.5 ± 2.7
CD20^+^CD32^+^ (MFI)	56.2 ± 7.5	84.9 ± 15.5	71.2 ± 17.9	66.0 ± 11.3	72.2 ± 13.6	126.6 ± 13.8	65.2 ± 12.2	87.5 ± 13.4

NI, Non-infected macaques; CCC(-), Absence of Chronic Chagasic Cardiopathy; CCC(+), Mild Chronic Chagasic Cardiopathy; CCC(++), Moderate Chronic Chagasic Cardiopahty. Data are expressed as mean values (% or mean fluorescence intensity-MFI) standard error. Multiple comparisons amongst groups were carried out by Kruskal-Wallis test followed by Dunn’s post-test for sequential pairwise comparisons and significant differences at p < 0.05 depicted by letters “a”, “b”, “c” and “d” as compared to NI, CCC(-), CCC(+) and CCC(++), respectively. All significant differences are highlighted in bold format.

The phenotypic profiles of innate and adaptive immunity cells in peripheral blood from *T. cruzi*-infected cynomolgus macaques and non-infected controls were further analyzed as biomarker signatures according to Luiza-Silva et al. (2011) and data are presented in **Figure 1**. For this purpose, the original flow cytometric measurements expressed in percentage or mean fluorescence intensity (MFI) were converted into categorical data and the results expressed as proportion of cynomolgus macaques with biomarker levels above the global median cut-off as described in Methods. The results are presented in **Figure 1**. Comparative analyses were carried out to identify in infected groups those biomarkers with significantly increased (*) or decreased (#) proportion, in relation to the proportions observed in non-infected controls (NI).

The results revealed lower proportion of several innate immunity cell phenotypes in peripheral blood of *T. cruzi*-infected macaques, by comparison with non-infected controls. However, higher frequency of monocytes (CD14^+^CD32^+^), NK-cells (CD16^+^CD54^+^) and NKT-cell subsets (CD3^+^CD16^+^CD56^+^; CD3^+^CD16^-^CD56^+^) were observed in asymptomatic/CCC(-) hosts; and higher frequencies of CD16^+^GranA^+^ cells were observed in hosts with mild/CCC(+) or moderate/CCC(++) chronic chagasic cardiopathy. In addition, a higher frequency of CD14^+^CD32^+^ cells was observed in hosts with moderate/CCC(++) chronic chagasic cardiomyopathy (**Figure 1**).

The analyses of the adaptive immunity in the peripheral blood also revealed significant reductions in frequencies of some cell phenotypes in *T. cruzi*-infected macaques as compared to controls, particularly in those hosts with moderate/CCC(++) chronic chagasic cardiopathy. Conversely, increased frequencies of some T-cell subsets (CD3^+^CD8^+^; CD3^+^CD54^+^; CD3^+^CCR5^+^; CD3^+^HLA-DR^+^; CD4^+^CD54^+^; CD4^+^CCR5^+^; CD4^+^CD25^++^; CD8^+^Perf^+^; CD8^+^GranB^+^) and B-cells (CD20^+^; CD20^+^CD32^+^) were observed in asymptomatic/CCC(-) hosts. Noteworthy was the increase in frequencies of activated T-cells (CD3^+^CD54^+^; CD3^+^HLA-DR^+^; CD3^+^CCR5^+^; CD4^+^CD54^+^; CD8^+^CD54^+^; CD8^+^ HLA-DR^+^; CD8^+^GranA^+^), along with CD20^+^ B-cells, in hosts with mild/CCC(+) chronic chagasic cardiopathy. Increased frequencies of T-cell subsets (CD3^+^CD8^+^; CD4^+^CD54^+^; CD4^+^CD25^++^; CD8^+^CD54^+^), along with CD20^+^CD32^+^ B-cells, were observed in hosts with moderate/CCC(++) chagasic cardiopathy (**Figure 1**).

### Analyses of Splenocytes From *T. cruzi*-Infected Cynomolgus Macaques Classified According to Histopathological Features of Chronic Chagasic Cardiopathy

Detailed flow cytometric results expressed as percentage or mean fluorescence intensity (MFI), together with descriptive statistical analysis for the phenotypic features of splenocytes from *T. cruzi*-infected macaques as compared to non-infected controls are presented in **Table 1**. Data analysis did not demonstrate any significant differences amongst groups based on conventional statistical analysis.

The phenotypic profiles of innate and adaptive immunity cells in peripheral blood from *T. cruzi*-infected cynomolgus macaques and non-infected controls were further analyzed as biomarker signatures according to Luiza-Silva et al. (2011) as described in Methods and data are presented in **Figure 2**. The phenotypic profiles of innate and adaptive immunity cells in the spleen compartment from *T. cruzi*-infected cynomolgus macaques and non-infected controls are presented in **Figure 2**. As for the analyses of peripheral blood leukocytes, the results are presented as biomarker signatures and comparative data analyses that indicate significantly increased (*) or decreased (#) proportion, in relation to the proportions in non-infected controls (NI).

As observed in peripheral blood biomarker signatures, the spleen compartment also displayed perturbations in frequencies of innate immunity cells in *T. cruzi*-infected macaques. Increased frequencies of NK-cells (CD3^-^CD16^+^; CD16^+^CD56^+^; CD16^+^CD56^-^; CD16^+^CD69^+^), and NKT-cell (CD3^+^CD16^+^CD56^+^) subsets were observed in asymptomatic/CCC(-) hosts. Increased frequencies of CD16^+^CD69^+^, CD16^+^CD56^-^, CD16^+^GranA^+^, and CD14^+^CD32^+^ cells were observed in hosts with mild/CCC(+) or moderate/CCC(++) chronic chagasic cardiopathy, respectively (**Figure 2**).

The adaptive immunity cell profile of the spleen compartment, like that of the biomarkers signature of blood compartment, also was perturbed in *T. cruzi*-infected macaques. Increased frequencies of T-cell subsets (CD3^+^; CD3^+^HLA-DR^+^; CD4^+^CD54^+^; CD4^+^CCR5^+^; CD4^+^CD25^++^) and B-cells (CD20^+^CD32^+^) were observed in asymptomatic/CCC(-) hosts. Increased frequencies of activated T-cells (CD3^+^CD69^+^; CD4^+^CD69^+^; CD8^+^CD69^+^; CD8^+^GranA^+^) were observed in hosts with mild/CCC(+) chronic chagasic cardiopathy. Noteworthy, increased frequencies of activated T-cell subsets (CD3^+^CD69^+^; CD3^+^HLA-DR^+^; CD4^+^CD25^++^; CD8^+^HLA-DR^+^; CD8^+^GranA^+^; CD8^+^GranB^+^), along with CD20^+^CD32^+^ B-cells, were observed in hosts with moderate/CCC(++) chagasic cardiopathy (**Figure 2**).

### *Ex Vivo* Cytokine Signatures of Peripheral Blood Leukocytes From *T. cruzi*-Infected Cynomolgus Macaques Classified According to Histopathological Features of Chronic Chagasic Cardiopathy

Detailed flow cytometric results expressed as per mile, together with descriptive statistical analysis for the functional cytokine profile of peripheral blood from *T. cruzi*-infected macaques as compared to non-infected controls are presented in **Table 2**. Data analysis demonstrated significant differences in cytokine profile of innate (Monocytes and NK-cells) and adaptive immunity (CD4^+^ T-cells, CD8^+^ T-cells and B-cells) of *T. cruzi*-infected cynomolgus macaques as compared to non-infected controls. Of note, higher levels of cytokine^+^ cells were observed in hosts with moderate/CCC(++) chronic chagasic cardiopathy ad compared to asymptomatic/CCC(-) hosts and non-infected controls (**Table 2**).

**Table 2:** *Ex vivo* cytokine profile of peripheral blood leukocytes from *T. cruzi*-infected cynomolgus macaques.

Cell Phenotypes	Peripheral Blood			
	NI	CCC(-)	CCC(+)	CCC(++)
Innate Immunity				
TNF^+^CD14^+^	24.6 ± 5.5	31.6 ± 4.7	31.9 ± 10.2	**17.4 ± 6,6^b^**
TNF^+^CD14^+^CD16^+^DR^++^	71.0 ± 46	60.8 ± 24.6	69.1 ± 7.8	61.8 ± 17.1
TNF^+^CD16^+^	12.0 ± 5.1	13.1 ± 4.0	**20.0 ± 8.2^d^**	7.6 ± 2.0
IFN^+^CD16^+^	17.6 ± 5.3	14.7 ± 2.5	14.1 ± 4.5	14.7 ± 3.1
IL-10^+^CD14^+^	31.3 ± 8.6	27.0 ± 8.2	24.4 ± 3.4	22.2 ± 4.8
IL-10^+^CD14^+^CD16^+^DR^++^	29.8 ± 8.2	27.1 ± 1.1	**14.2 ± 2.5^b,d^**	37.5 ± 7.8
Adaptive Immunity				
TNF^+^CD4^+^	6.8 ± 1.4	7.5 ± 1.9	4.5 ± 1.6	7.0 ± 1.5
TNF^+^CD8^+^	4.9 ± 1.0	4.5 ± 0.7	3.0 ± 1.0	**6.6 ± 1.0^c^**
TNF^+^CD20^+^	7.6 ± 1.4	7.3 ± 1.8	7.3 ± 2.2	**14.5 ± 9.6^a^**
IFN^+^CD4^+^	4.8 ± 0.7	**9.0 ± 1.3 ^a^**	**8.3 ± 1.3^a^**	**10.4 ± 2.2^a^**
IFN^+^CD8^+^	5.0 ± 1.0	2.8 ± 0.7	3.5 ± 1.1	**5.7 ± 2.2^a,b^**
IL-10^+^CD4^+^	6.5 ± 2,4	**11.1 ± 2.6 ^a^**	**17.0 ± 4.4^a^**	**11.5 ± 2.6^a^**
IL-10^+^CD8^+^	15.1 ± 2.9	9.7 ± 2.6	**23.0 ± 4.6^b^**	**32.1 ± 8.1^a,b^**
IL-10^+^CD20^+^	25.0 ± 8.7	23.4 ± 3.0	24.7 ± 6.6	**40.0 ± 14.9^a^**

NI, Non-infected macaques; CCC(-), Absence of Chronic Chagasic Cardiopathy; CCC(+), Mild Chronic Chagasic Cardiopathy; CCC(++), Moderate Chronic Chagasic Cardiopahty. Data are expressed as mean values (‰) ± standard error. Multiple comparisons amongst groups were carried out by Kruskal-Wallis test followed by Dunn’s post-test for sequential pairwise comparisons and significant differences at p<0.05 depicted by letters “a”, “b”, “c” and “d” as compared to NI, CCC(-), CCC(+) and CCC(++), respectively. All significant differences are highlighted in bold format.

**Figure 3:**
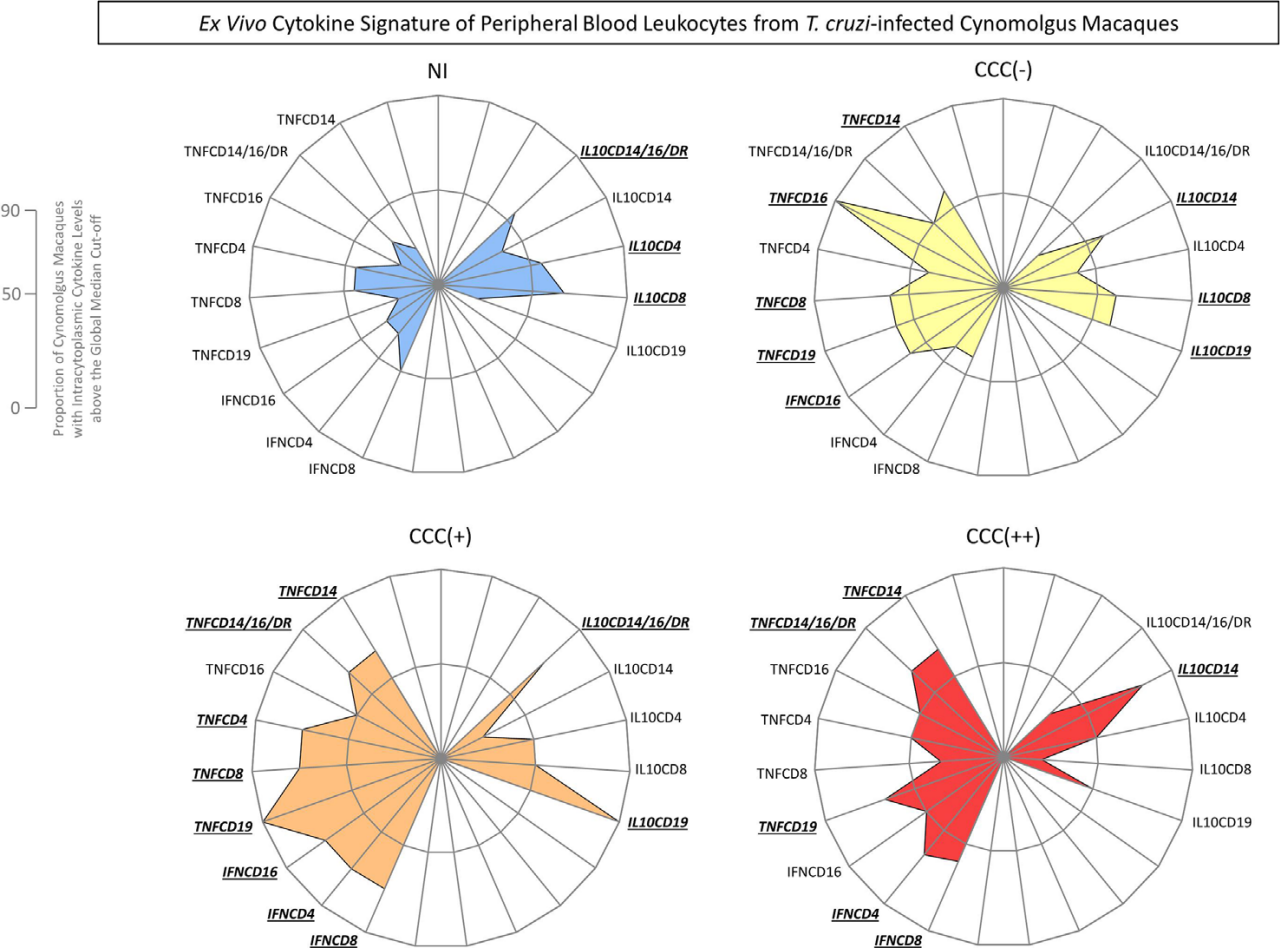
*Ex vivo* cytokine signatures of peripheral blood leukocytes from *T. cruzi*-infected cynomolgus macaques classified according to histopathological features of chronic chagasic cardiopathy. The functional cytokine signatures were constructed based on the proportion of subjects with intracytoplasmic cytokine levels above the global median cut-off defined for each cell subset, calculated for the entire study population. Radar charts were built to obtain the overall profile of pro-inflammatory (left side) and regulatory (right side) cytokines for innate and adaptive immunity cells. Distinct color backgrounds were used to tag the non-infected controls (NI = 
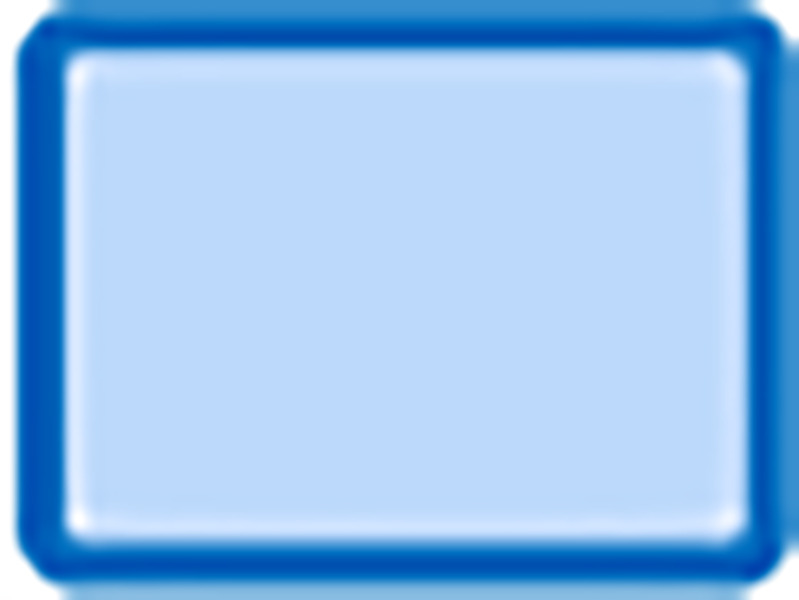
 ) and the subgroups of *T. cruzi*-infected cynomolgus macaques, classified according to histopathological features of cardiac biopsies and referred to as CCC(–) for absence of chronic chagasic cardiopathy (
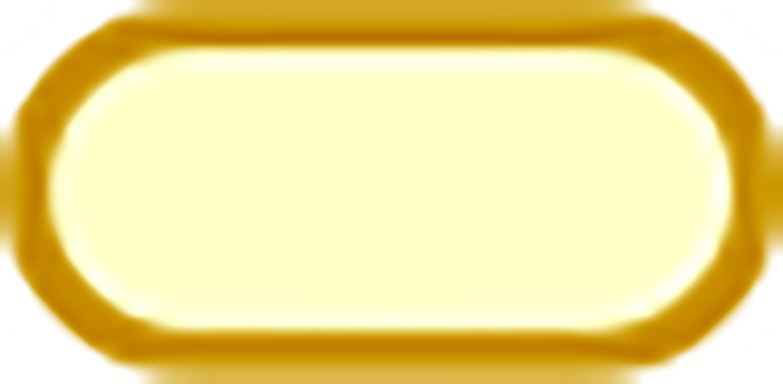
); CCC (+) for mild chronic chagasic cardiopathy (
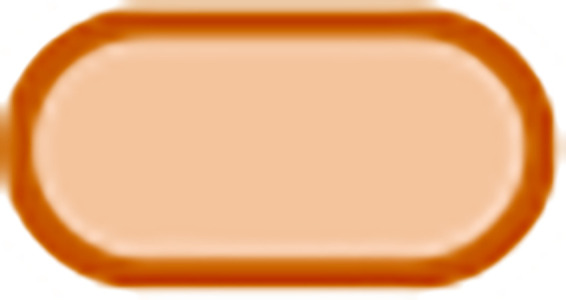
) and CCC (++) for moderate chronic chagasic cardiopathy (
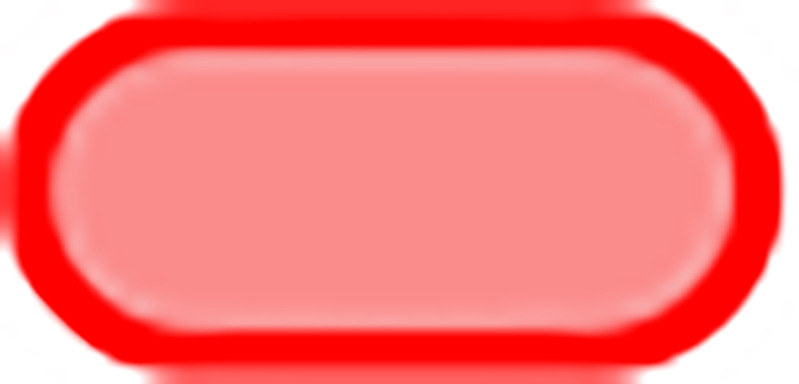
). The biomarkers with frequencies above the 50^th^ percentile were highlighted for each group by bold underline format.

The *ex vivo* functional profiles of innate and adaptive immunity cells from peripheral blood from *T. cruzi*-infected cynomolgus macaques and non-infected controls were further analyzed as biomarker signatures according to Luiza-Silva et al. (2011) and data are presented in **Figure 3**. The results are presented in radar charts as cytokine signatures depicting the proportion of subjects with intracytoplasmic cytokine levels above the global median cut-off calculated for each cell subset.

Data analyses demonstrated that a balanced cytokine microenvironment mediated by TNF-α (TNFCD14; TNFCD16; TNFCD8; TNFCD19) and IFN-*γ* (IFNCD16) counterbalanced by IL-10 (IL10CD14; IL10CD8; IL10CD19) was the hallmark of asymptomatic/CCC(-) hosts. On the other hand, a predominant pro-inflammatory profile triggered by TNF-α (TNFCD14; TNFCD14/16/DR; TNFCD4; TNFCD8; TNFCD19) and IFN-*γ* (IFNCD16; IFNCD4; IFNCD8) with minor increased production of IL-10 (IL10CD14/16/DR; IL10CD19) was observed in hosts with mild/CCC(+) chagasic cardiopathy. Furthermore, a prominent pro-inflammatory profile generated by TNF-α (TNFCD14; TNFCD14/16/DR; TNFCD19) and IFN-*γ* (IFNCD4; IFNCD8) with a slight contribution of IL-10 (IL10CD14) was identified in hosts with moderate/CCC(++) chagasic cardiopathy (**Figure 3**).

### Set of Phenotypic/Functional Biomarkers Useful to Depict the Cardiac Lesion Status in *T. cruzi*-Infected Cynomolgus Macaques

**Figure 4:**
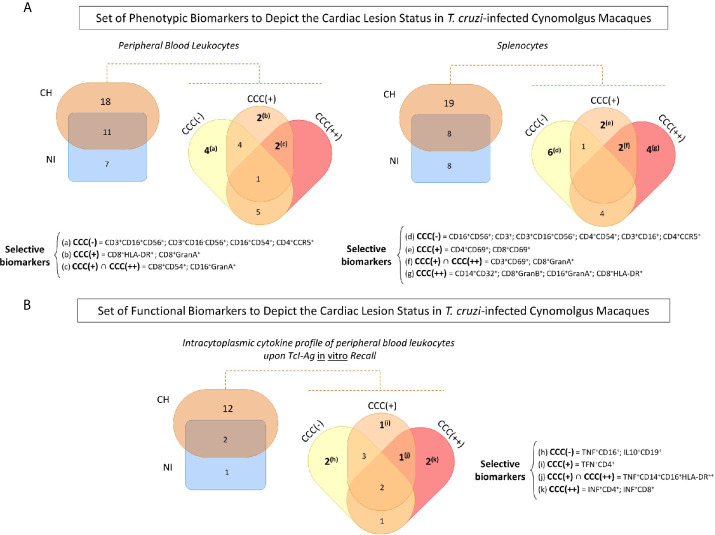
Set of phenotypic/functional biomarkers useful to depict the cardiac lesion status in *T. cruzi*-infected cynomolgus macaques. **(A)** Venn diagram analyses were carried out to identify common and selective phenotypic biomarkers in peripheral blood and spleen samples from *T. cruzi*-infected cynomolgus macaques (CH = 
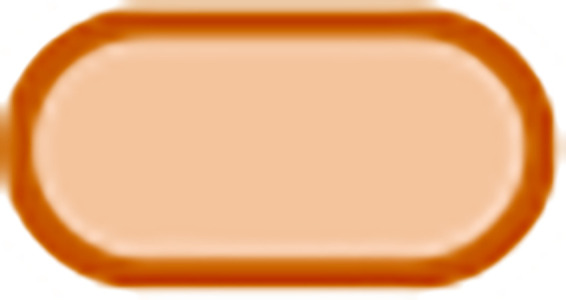
 ) and non-infected controls (NI = 
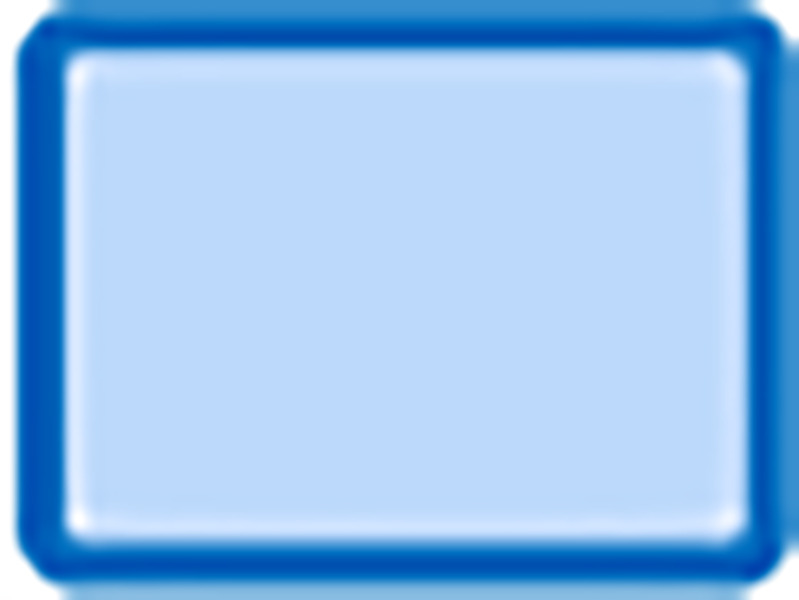
), and subsequently among subgroups of *T. cruzi*-infected macaques, classified according to histopathological features of cardiac biopsies and referred as CCC (–) for absence of chronic chagasic cardiopathy (
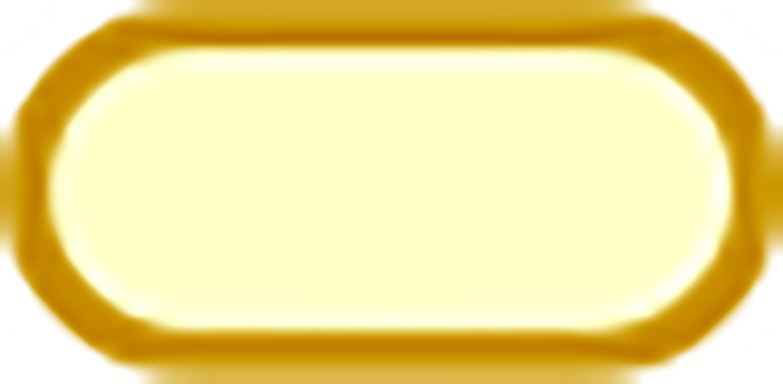
); CCC (+) for mild chronic chagasic cardiopathy (
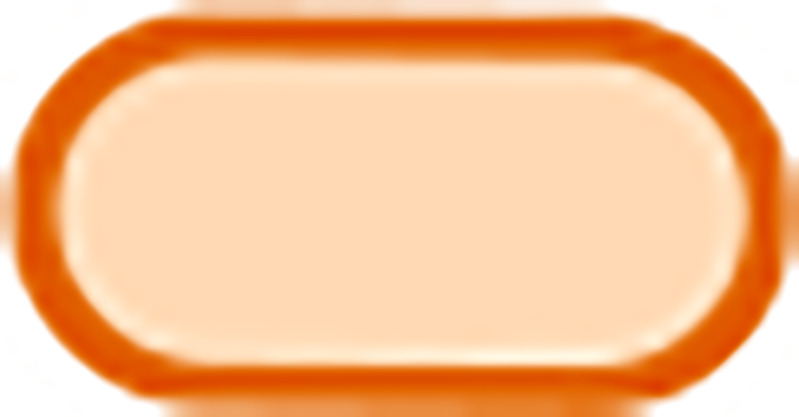
) and CCC (++) for moderate chronic chagasic cardiopathy (). **(B)** Venn diagram analyses were performed to select, within the intracytoplasmic cytokine profile of peripheral blood leukocytes upon TcI-Ag recall *in vitro*, the common and unique functional biomarkers for *T. cruzi*-infected cynomolgus macaques (CH) and non-infected controls (NI) and subsequently among the CCC (–), CCC (+) and CCC (++) groups. The selected sets of biomarkers are shown in the figure. Detailed data and conventional statistical analysis is presented in **Supplementary Table 1**.

Venn diagram analyses were carried out to identify sets of biomarkers differentially observed in *T. cruzi*-infected cynomolgus macaques and non-infected controls, as well as sets differentially observed among subgroups of *T. cruzi*-infected animals classified according to histopathological features of cardiac biopsies. The data are presented in **Figure 4** and **Supplementary Table 1**.

Analysis of peripheral blood leukocytes revealed 11 biomarkers common to *T. cruzi*-infected cynomolgus macaques (CH) and non-infected controls (NI), 7 biomarkers that were distinct for the NI group, and 18 biomarkers that were distinct for the CH group (**Supplementary Table 1**). Four biomarkers were the hallmark of asymptomatic/CCC(-) hosts (CD3+CD16+CD56+; CD3+CD16^-^CD56^+^; CD16^+^CD54^+^; CD4^+^CCR5^+^); two biomarkers were commonly observed in hosts with chronic chagasic cardiopathy, including CD16^+^GranA^+^ and CD8^+^CD54^+^; and CD8^+^HLA-DR^+^ and CD8^+^GranA^+^ were selectively observed in hosts with mild/CCC(+) chronic chagasic cardiopathy.

In the spleen compartment, Venn Diagram analysis demonstrated that 8 common biomarkers were identified in *T. cruzi*-infected cynomolgus macaques (CH) and non-infected controls (NI). Sets of 19 and 8 biomarkers were discriminatory for CH and NI, respectively (**Supplementary Table 1**). Six biomarkers were selectively found in asymptomatic/CCC(-) hosts (CD16^+^CD56^+^; CD3^+^CD16^+^; CD3^+^CD16^+^CD56^+^; CD3^+^; CD4^+^CD54^+^; CD4^+^CCR5^+^). Two phenotypic feature were commonly observed in hosts with chronic chagasic cardiopathy, including CD3^+^CD69^+^ and CD8^+^GranA^+^ while CD4^+^CD69^+^ and CD8^+^CD69^+^ were observed in hosts with mild/CCC(+) chronic chagasic cardiopathy. Furthermore, four biomarkers (CD14^+^CD32^+^; CD16^+^GranA^+^; CD8^+^GranB^+^; CD8^+^HLA-DR^+^) were selectively found in hosts with moderate/CCC(++) chronic chagasic cardiopathy (**Figure 4A** and **Supplementary Table 1**).

Sets of functional biomarkers were also identified to depict the cardiac lesion status in *T. cruzi*-infected cynomolgus macaques. Data demonstrated that 12 functional features were able to discriminate *T. cruzi*-infected cynomolgus macaques (CH) from non-infected controls (NI) (**Supplementary Table 1**). Two biomarkers were selectively observed in asymptomatic/CCC(-) hosts (TNF^+^CD16^+^; IL10^+^CD19^+^), underscoring a balanced cytokine profile. On the other hand, a clear pro-inflammatory profile was observed in hosts with chronic chagasic cardiopathy, mediated by TNF^+^CD14^+^CD16^+^HLA-DR^++^. Additionally, TFN^+^CD4^+^ counts for the pro-inflammatory milieu observed in hosts with mild/CCC(+) chronic chagasic cardiopathy, whereas INF^+^CD4^+^; INF^+^CD8^+^ further contribute for the exacerbated inflammatory microenvironment in hosts with moderate/CCC(++) chronic chagasic cardiopathy (**Figure 4B** and **Supplementary Table 1**).

## Discussion

Non-human primates are recognized models for studying a wide range of human infectious diseases. In fact, the study of natural or experimental infectious diseases in non-human primates has enabled the development of improved vaccines, diagnostic tools, and therapeutic strategies for human diseases (Gardner and Luciw, 2008). Cynomolgus macaques have been suggested as useful models for studies on Chagas disease, based on PCR assessment of *T. cruzi* in cardiac tissue, histopathological features, and electrocardiograms (Zabalgoitia et al., 2003; Williams et al., 2009; Padilla et al., 2021), as well as phenotypic and functional features of immune response that these animals share in common with humans after natural infection with *T. cruzi* (Sathler-Avelar et al., 2016; Vitelli-Avelar et al., 2017; Padilla et al., 2021). Aiming at further understanding the immune response of cynomolgus macaques that were naturally infected with *T. cruzi*, the present investigation enabled a detailed immunophenotypic and functional analysis of peripheral blood and spleen cells, and identification of their unique and shared features in relation to cardiac histopathological lesion status. For this purpose, the animals were classified as CCC(-), CCC(+) and CCC(++), designating their diagnosis of asymptomatic, mild or moderate chronic chagasic cardiopathy, respectively. Overall, our data demonstrated that CCC(-) macaques displayed increased levels of circulating and splenic monocytes, NK cells and NKT cells, by comparison with non-infected animals, suggesting that these cells may play a role in protecting against heart disease. These findings are in agreement with those previously reported by Vitelli-Avelar and co-workers (Vitelli-Avelar et al., 2005; Vitelli-Avelar et al., 2006), showing that asymptomatic, indeterminate Chagas disease patients exhibited an immune response profile characterized by increased levels of circulating proinflammatory monocytes (CD14^+^CD16^+^HLA‐DR^++^), and a high frequency of NKT-cells (CD3^+^CD16^-^CD56^+^) along with an elevated frequency of NK-cells (CD3-CD16+CD56+ and CD3-CD16+CD56dim). A comparative analysis of these immunophenotypes in asymptomatic children (Vitelli-Avelar et al., 2006) and patients with late chronic indeterminate form of Chagas disease (Vitelli-Avelar et al., 2005) suggested that a shift of circulating leukocytes toward high values of macrophage‐like cells as well as a high frequency of NK-cells and NKT-cells are associated with limited tissue damage and the establishment/maintenance of a lifelong stable and asymptomatic form of chronic Chagas disease. The protective role of monocytes in asymptomatic Chagas disease has been already postulated. It has been shown that *in vitro T. cruzi*-infection of monocytes from indeterminate patients led to a decreased expression of HLA-DR, but increased expression of CD80 (Souza et al., 2004). While lower HLA-DR expression contributes to maintain T-cell activation at low levels, the increased level of CD80, a ligand for CTLA-4 which is up-regulated on T-cells from indeterminate patients, is likely to contribute for the modulation of T-cell response in asymptomatic patients (Dutra et al., 2009).

Our results also demonstrated that a robust adaptive cell‐mediated inflammatory response, characterized by increased levels of CD8^+^ activated T-cells, along with a high frequency B-cells, are the hallmarks of hosts with mild/CCC(+) and moderate/CCC(++) chronic chagasic cardiopathy. High levels of circulating activated CD8^+^ T-cells have been reported in patients with late cardiac Chagas disease. These data re-enforce that strong activation of CD8^+^ T-cells could lead to tissue damage and the development of cardiomyopathy in Chagas disease. In fact, Reis and colleagues (Reis et al., 1993) have shown that these cells, many of which express Granzyme A, are predominant in cardiac tissues from patients with severe chronic chagasic cardiomyopathy. Only a few macrophage-like monocytes and small numbers of NK-cells or B lymphocytes were reported in cardiac lesions (Reis et al., 1993). These findings in human Chagas disease support the hypothesis that CD8^+^ T-cells play an immunopathological role in Chagas disease. Previous studies have demonstrated that activated CD8^+^ T-cells can also be observed in some patients with indeterminate Chagas disease (Dutra et al., 1994). However, it has been shown that CD8^+^ T-cells from indeterminate patients displayed an up-regulated expression of CTLA-4 (Souza et al., 2007), suggesting that these cells may be self-regulated, possibly due to intrinsic regulation *via* CTLA-4 (Dutra et al., 2009).

Increased levels of CD4^+^CCR5^+^ T-cells were observed in the asymptomatic/CCC(-) macaque hosts. CCR5 is considered to be a classic pro-inflammatory chemokine receptor, preferentially expressed by NK-cells, macrophages, antigen-presenting cells, activated and effector memory T-cells, but also expressed by regulatory T-cells (Bonecchi et al., 1998; Huehn and Hamann, 2005; Scurci et al., 2018). It has been shown that, at sites of infection or tissue damage, CCR5 ligands recruit the ingress and activation of effector cells to release chemokines and to further amplify the pro-inflammatory cascade (Bachelerie et al., 2014). However, CCR5 stimulation may also modulate the activation, behavior and survival of immunity cells in tissues (Kohlmeier et al., 2011). Therefore, the involvement of CCR5 in the recruitment of regulatory T-cells (Huehn and Hamann, 2005) indicates a dual role for this receptor, not only inducing but also resolving inflammatory response. Previous studies have tried to decipher whether CCR5 plays a role in the development of cardiac injuries or if it is a protective biomarker in Chagas disease (Talvani et al., 2004; Nogueira et al., 2012; de Oliveira et al., 2016; Miranda et al., 2017; Roffe et al., 2019). CCR5^+^ T-cells have been found in association with *T. cruzi* nests and antigens in heart tissue during murine acute infection, suggesting a direct anti-parasitic role (Marino et al., 2004) as well as its involvement in immunopathological mechanisms (Marino et al., 2005). Nogueira and colleagues (Nogueira et al., 2012) demonstrated CCR5 expression on mononuclear cells in the myocardium of cardiac patients, but a comparative analysis was not carried out on biopsies from indeterminate asymptomatic patients. Miranda and colleagues (Miranda et al., 2017) did not find differences in the percentages of CCR5^+^ T-cells, both CD4^+^ and CD8^+^, in peripheral blood from patients with different clinical forms of Chagas disease. Consistent with our findings, a previous study has shown a correlation between the CCR5 expression and the degree of heart function, such that the more severe the chronic chagasic cardiomyopathy, the lower the expression of CCR5 by circulating CD4^+^ and CD8^+^T-cells (Talvani et al., 2004). The expression of CCR5 by T-cells has been recently addressed by Roffe and colleagues (Roffe et al., 2019) showing that the percentage of effector and effector memory CCR5^+^ T-cells, both CD4^+^ and CD8^+^, were increased in patients with cardiac Chagas disease. Being somewhat controversial, the role of chemokine receptor CCR5 in the pathogenesis of cardiac Chagas disease needs to be investigated further. More studies are required to identify the exact role of this chemokine receptor in *T. cruzi*-induced heart injury and also in distinct clinical forms of Chagas disease.

The immunophenotypic profiles associated with histopathological characteristics in *T. cruzi*-infected cynomolgus macaques resemble those observed in human Chagas disease, demonstrating that besides developing comparable histopathological features (Gardner and Luciw, 2008; Williams et al., 2009), these animals also have a similar immune response in relation to the distinct clinical forms (Vitelli-Avelar et al., 2005; Vitelli-Avelar et al., 2006; Dutra et al., 2009). It is well established that *T. cruzi* infection simultaneously elicits multiple functional events of innate and adaptive immunity, leading to systemic production of pro-inflammatory and regulatory cytokines. This complex microenvironment requires the participation of distinct cell phenotypes throughout the activation of innate immune responses, mediated by NK-cells and macrophages in conjunction with adaptive immunity, involving distinct T-cell subsets (Dutra et al., 2009; Dutra et al., 2014). In this sense, our results demonstrated that there was a typical pro-inflammatory/anti-inflammatory immune-modulated profile in CCC(-) hosts, mediated by a mixed TNF/IFN/IL-10 cytokine milieu. Conversely, hosts with mild/CCC(+) or moderate/CCC(++) chronic chagasic cardiopathy exhibited a predominant pro-inflammatory profile, with prominent production of TNF and IFN. These findings are in agreement with those previously reported for human patients with distinct clinical forms of Chagas disease (Teixeira-Carvalho et al., 2002; Gomes et al., 2003; Dutra et al., 2009; Sathler-Avelar et al., 2012; Dutra et al., 2014), demonstrating that this pattern characteristic in Chagas disease of primate species.

The functional aspects of distinct cell subsets have been extensively investigated in regard to the induction or modulation of immunopathology in clinical forms of Chagas disease (Reis et al., 1993; Corrêa-Oliveira et al., 1999; Vitelli-Avelar et al., 2005; Dutra et al., 2009; Dutra et al., 2014; Acevedo et al., 2018). Dutra and colleagues (Dutra et al., 2014) have published an extensive review of the immunoregulatory mechanisms involved in human *T. cruzi* infection, discussing the predominance of an anti-inflammatory milieu in indeterminate patients while an inflammatory profile is typically observed in the cardiac form of Chagas disease. It has been shown that different kinetics of cytokine production is relevant for determining the fate of Chagas disease. It is well known that, while pro-inflammatory cytokines, such as TNF and IFN-*γ*, are relevant to trigger immunological mechanisms to control the parasite growth; the establishment of immunomodulatory events, mediated by IL-10, is essential to prevent disease morbidity. In fact, this balance requires fine tuning between the over production of pro-inflammatory cytokines and the production of IL-10 to prevent an immunosuppressive effect on the cellular response, enough to allow the control of the parasite, but not so much as to cause tissue damage (Dutra et al., 2014).

Classical studies have proposed that the phenotypic and functional aspects of peripheral blood leukocytes population observed in *T. cruzi*-infected hosts are similar to those found at the tissue level (Reis et al., 1993; Higuchi et al., 2003; Fonseca et al., 2007; Vitelli-Avelar et al., 2008; Dutra et al., 2009; Cunha-Neto and Chevillard, 2014). In the present study we did not have the opportunity to characterize the cardiac inflammatory infiltrate by Immunohistochemistry analysis. However, it has been previously demonstrated that cynomolgus macaques naturally infected with *T. cruzi* exhibited mild to moderate multifocal areas of inflammatory infiltrates that were composed mainly of mononuclear cells with fewer neutrophils. The immunohistochemistry revealed that the mononuclear cells were predominantly CD8^+^ and CD68^+^ with fewer CD4^+^ lymphocytes (Pisharath et al., 2013), indicating an inflammatory profile similar to that observed in humans (Reis et al., 1993; Higuchi et al., 2003).

In summary, we have presented a broad analysis of several phenotypic and functional aspects of peripheral blood leukocytes and spleen cells from cynomolgus macaques that were naturally infected with *T. cruzi*, in relation to cardiac histopathological characteristics. Altogether our data revealed that cynomolgus macaques display histological features that are associated with particular profiles of immune response similarly to those observed in humans. These similarities further sustain the employment of cynomolgus macaques in pre-clinical research on Chagas disease and provide insights about the mechanisms implicated in the development and maintenance of chagasic heart disease.

## Data Availability Statement

The original contributions presented in the study are included in the article/**Supplementary Material**. Further inquiries can be directed to the corresponding authors.

## Ethics Statement

The animal study was reviewed and approved by Texas Biomedical Research Institute Animal Care and Use Committee (#1050MF).

## Author Contributions

Conceptualization: RS-A, DV-A, SE-S, AT-C, JFV, JLV, and OM-F. Funding acquisition: JFV, JLV, and OM-F. Investigation: RS-A, DV-A, and ED. Methodology: RS-A, DV-A, AM-B, MX, ED, and OM-F. Data analysis: RS-A, DV-A, AM-B, MX, ED, and OM-F. Data curation: RS-A and DV-A. Project administration: JFV and JLV. Supervision: JFV, JLV, and OM-F. Writing original draft, review & editing: RS-A, DV-A, AM-B, MX, SE-S, IC-R, JFV, JLV, and OM-F. All authors contributed to the article and approved the submitted version.

## Funding

This study was supported by the Southwest National Primate Research Center’s NIH base grant (5 P51 RR013986), the European Community’s Seventh Framework Program (No. 602773 – Project KINDRED), IRR/Fundação Oswaldo Cruz (FIOCRUZ-Minas), Fundação de Amparo à Pesquisa de Minas Gerais (FAPEMIG), Conselho Nacional de Desenvolvimento Científico e Tecnológico (CNPq) and Coordenação de Aperfeiçoamento de Pessoal de Nível Superior (CAPES). ATC and OAMF thank CNPq for fellowships (PQ/CNPq). OM-F is a research fellow from FAPEAM (PVN-II, PRÓ-ESTADO Program #005/2019). The funders had no role in study design, data collection and analysis, decision to publish or preparation of the manuscript.

## Conflict of Interest

The authors declare that the research was conducted in the absence of any commercial or financial relationships that could be construed as a potential conflict of interest.

## Supplementary Material

Supplementary Figure 1 Flow cytometry gating strategy. *Ex vivo* immunophenotypic analysis of peripheral blood leukocytes **(A)** and splenocytes **(B)** were carried out by flow cytometry as described in Material and Methods. Lymphocytes were first gated based on their size and granularity properties using pseudocolor plots of Forward Scatter (FSC) *vs* Side Scatter (SSC). CD3^+^ T-cells were further selected within gated lymphocytes. Following, CD4^+^ and CD8^+^ T-cells were gated within CD3^+^ T-cells. Phenotypic features of CD4^+^ and CD8^+^ T-cells were then analyzed on bidimensional pseudocolor plots (e.g. CCR5^+^, Gran-A^+^, CD69^+^ and Gran-B^+^). *Ex vivo* functional analysis strategy of peripheral blood leukocytes **(C)** were also assessed by intracytoplasmic immunophenotyping. Total lymphocytes were first gated on a Forward Scatter (FSC) *vs* Side Scatter (SSC) plot, followed by CD3^+^ T-cells and CD4^+^ and CD8^+^ T-cell subsets gating. Selection of CD20^+^ B-cells was also carried out within gated lymphocytes. Functional features cell subsets were quantified as cytokine^+^ events on bidimensional pseudocolor plots (e.g. TNF-α^+^, IFN-*γ* ^+^, and IL-10^+^).
