# Supplementary figures and images for: Phenotypic and Functional Signatures of Peripheral Blood and Spleen Compartments of Cynomolgus Macaques Infected With T. cruzi: Associations With Cardiac Histopathological Characteristics
Source: Front Cell Infect Microbiol. 2021 Jul 14;11:701930. doi: 10.3389/fcimb.2021.701930 (PMC8317693; doi:10.3389/fcimb.2021.701930)

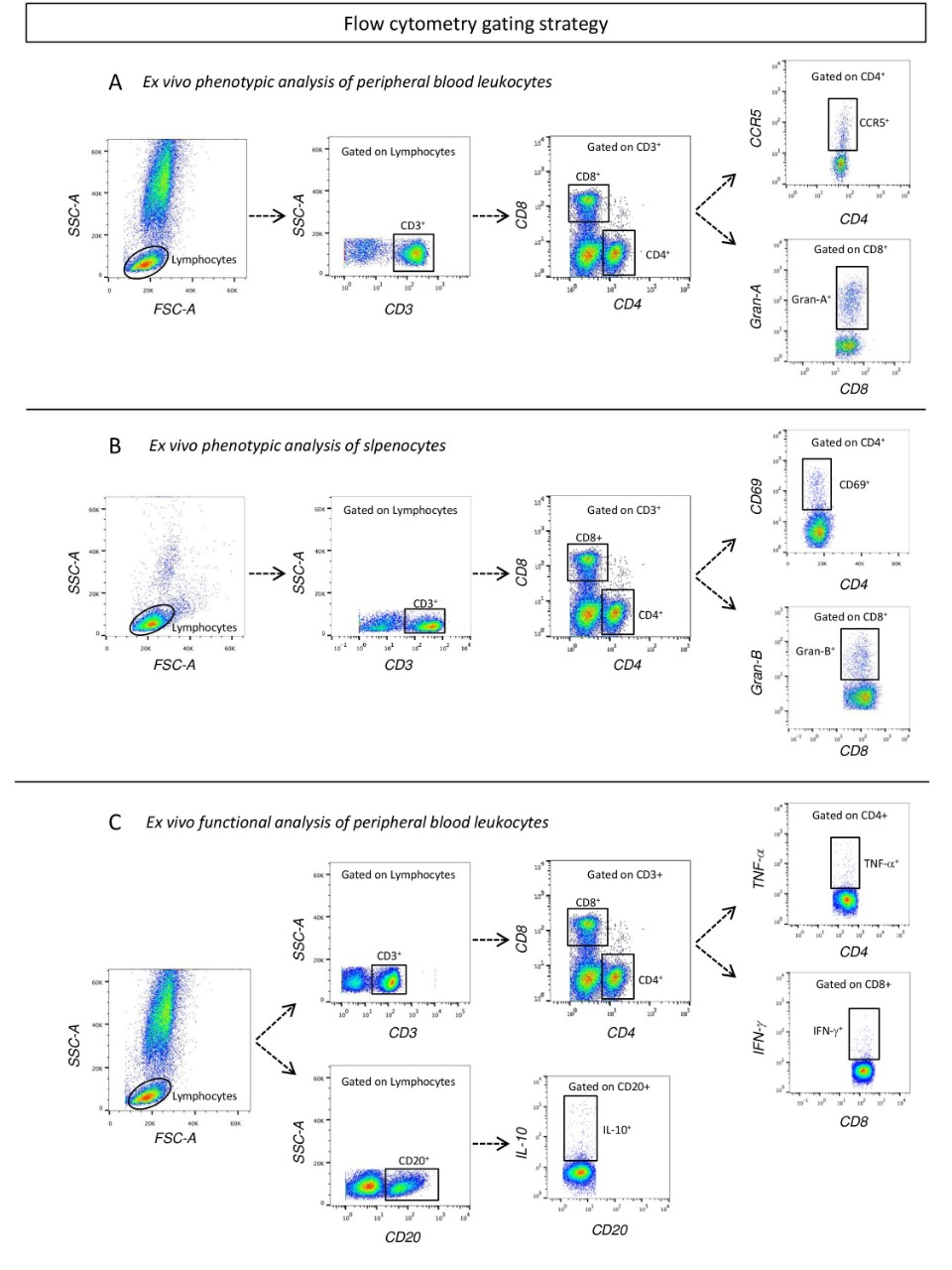

Supplement: Supplementary Figure 1 — Flow cytometry gating strategy. Ex vivo immunophenotypic analysis of peripheral blood leukocytes (A) and splenocytes (B) were carried out by flow cytometry as described in Material and Methods. Lymphocytes were first gated based on their size and granularity properties using pseudocolor plots of Forward Scatter (FSC) vs Side Scatter (SSC). CD3+ T-cells were further selected within gated lymphocytes. Following, CD4+ and CD8+ T-cells were gated within CD3+ T-cells. Phenotypic features of CD4+ and CD8+ T-cells were then analyzed on bidimensional pseudocolor plots (e.g. CCR5+, Gran-A+, CD69+ and Gran-B+). Ex vivo functional analysis strategy of peripheral blood leukocytes (C) were also assessed by intracytoplasmic immunophenotyping. Total lymphocytes were first gated on a Forward Scatter (FSC) vs Side Scatter (SSC) plot, followed by CD3+ T-cells and CD4+ and CD8+ T-cell subsets gating. Selection of CD20+ B-cells was also carried out within gated lymphocytes. Functional features cell subsets were quantified as cytokine+ events on bidimensional pseudocolor plots (e.g. TNF-α+, IFN-γ +, and IL-10+). [file Image_1.jpeg]
