# Supplementary material for: Phenotypic and Functional Signatures of Peripheral Blood and Spleen Compartments of Cynomolgus Macaques Infected With T. cruzi: Associations With Cardiac Histopathological Characteristics
Source: Front Cell Infect Microbiol. 2021 Jul 14;11:701930. doi: 10.3389/fcimb.2021.701930 (PMC8317693; doi:10.3389/fcimb.2021.701930)
Supplement: Supplementary file 2 [file Table_1.docx]

**Phenotypic and functional signatures of peripheral blood and spleen compartments of cynomolgus macaques infected with *T. cruzi*: Associations with cardiac histopathological characteristics** Renato Sathler-Avelar

Danielle Marquete Vitelli-Avelar

Armanda Moreira Mattoso-Barbosa

Marcelo Antônio Pascoal Xavier

Silvana Maria Elói-Santos

Andréa Teixeira-Carvalho

Edward J. Dick, Jr

Jane F. VandeBerg

John L. VandeBerg

Olindo Assis Martins-Filho

Supplementary Table S1. Detailed Venn diagram report of phenotypic/functional biomarkers useful to depict the cardiac lesion status in *T. cruzi*-infected cynomolgus macaques

| **Biomarkers** |  | **Groups** | **Set of Attributes** |
| --- | --- | --- | --- |
| **Phenotypic**  **Peripheral Blood Leukocytes** |  | **NI** | CD16^+^Perf^+^; CD16^+^CD56^+^ ; CD16^+^GranB^+^; CD16^+^CD69^+^; CD16^+^CD56^+^; CD3^-^CD16^+^; CD3^+^CD16^+^CD56^-^ |
|  |  | **NI ∩ CH** | CD3^+^CD16^+^; CD14^+^CD64^+^; CD16^+^CD56^+^; CD3^+^; CD3^+^CD4^+^; CD3^+^CD69^+^; CD4^+^CD69^+^; CD8^+^CD69^+^; CD4^+^HLA-DR^+^; CD8^+^CCR5^+^; CD20^+^CD69^+^ |
|  |  | **CH** |  |
|  |  | **CCC(-)** | CD16^+^CD54^+^; CD3^+^CD16^+^CD56^+^; CD3^+^CD16^-^CD56^+^; CD4^+^CCR5^+^ |
|  |  | **CCC(-) ∩ CCC(+)** | CD3^+^CD54^+^; CD3^+^HLA-DR^+^; CD3^+^CCR5^+^; CD20^+^ |
|  |  | **CCC(-) ∩ CCC(++)** | CD14^+^CD32^+^; CD3^+^CD8^+^; CD4^+^CD25^++^; CD8^+^GranB^+^; CD20^+^CD32^+^ |
|  |  | **CCC(-) ∩ CCC(+) ∩ CCC(++)** | CD4^+^CD54^+^ |
|  |  | **CCC(+)** | CD8^+^HLA-DR^+^; CD8^+^GranA^+^ |
|  |  | **CCC(+) ∩ CCC(++)** | CD16^+^GranA^+^; CD8^+^CD54^+^ |
| **Phenotypic**  **Splenocytes** |  | **NI** | CD14^+^CD64^+^; CD16^+^Pref^+^; CD16^+^GranB^+^; CD3^+^CD8^+^; CD3^+^CD54^+^; CD3^+^CCR5^+^; CD8^+^CD54^+^; CD20^+^ |
|  |  | **NI ∩ CH** | CD16^-^CD56^+^; CD16^+^CD54^+^; CD3^+^CD16^+^; CD3^+^CD16^+^CD56^-^; CD3^+^CD16^-^CD56^+^; CD3^+^CD4^+^; CD8^+^CCR5^+^; CD20^+^CD69^+^ |
|  |  | **CH** |  |
|  |  | **CCC(-)** | CD16^+^CD56^+^; CD3^+^CD16^+^; CD3^+^CD16^+^CD56^+^; CD3^+^; CD4^+^CD54^+^; CD4^+^CCR5^+^ |
|  |  | **CCC(-) ∩ CCC(+)** | CCD16^+^CD69^+^ |
|  |  | **CCC(-) ∩ CCC(++)** | CD16^+^CD56^+^; CD3^+^HLA-DR^+^; CD4^+^CD25^++^; CD20^+^CD32^+^ |
|  |  | **CCC(+)** | CD4^+^CD69^+^; CD8^+^CD69^+^ |
|  |  | **CCC(+) ∩ CCC(++)** | CD3^+^CD69^+^; CD8^+^GranA^+^ |
|  |  | **CCC(++)** | CD14^+^CD32^+^; CD16^+^GranA^+^; CD8^+^GranB^+^; CD8^+^HLA-DR^+^ |
| **Functional**  **Peripheral Blood Leukocytes upon TcI-Ag *in vitro* Recall** |  | **NI** | IL10^+^CD4^+^ |
|  |  | **NI ∩ CH** | IL10^+^CD14^+^CD16^+^HLA-DR^++^; IL10^+^CD8^+^ |
|  |  | **CH** |  |
|  |  | **CCC(-)** | TNF^+^CD16^+^; IL10^+^CD19^+^ |
|  |  | **CCC(-) ∩ CCC(+)** | IFN^+^CD16^+^; TNF^+^CD8^+^; IL10^+^CD19^+^ |
|  |  | **CCC(-) ∩ CCC(++)** | IL10^+^CD14^+^ |
|  |  | **CCC(-) ∩ CCC(+) ∩ CCC(++)** | TNF^+^CD14^+^; TNF^+^CD19^+^ |
|  |  | **CCC(+)** | TFN^+^CD4^+^ |
|  |  | **CCC(+) ∩ CCC(++)** | TNF^+^CD14^+^CD16^+^HLA-DR^++^ |
|  |  | **CCC(++)** | INF^+^CD4^+^; INF^+^CD8^+^ |

NI=Non-infected macaques; CH = *T. cruzi*-infected Cynomolgus macaques; CCC(-) = Absence of Chronic Chagasic Cardiopathy; CCC(+) = Mild Chronic Chagasic Cardiopathy; CCC(++) = Moderate Chronic Chagasic Cardiopahty. The biomarkers highlighted by underline format represent those selectively observed in subgroups of *T. cruzi*-infected Cynomolgus macaques.
